# An Anodyne in Use for Vesical Irritation

**Published:** 1887-09

**Authors:** 


**An Anodyne in Use for Vesical Irritation.**—Dr. W. P. Copeland, of Eufaula, Ala., writes as follows to the *Medical Record:* "In almost every community there are old men who suffer from enlarged prostates, accompanied with a chronic inflammation of the neck of the bladder, rendering them miserable sufferers, and a care and anxiety to their friends and families. Having had the professional care of several of this class of cases, and dreading the tendency they so frequently acquire to the administration of opium for the relief of pain, I resorted to various washes for injecting the bladder, resulting in my adopting a solution of benzoate of soda, ten grains to one ounce of water, with twenty to thirty drops of the green tincture of gelsemium; this is warmed and injected by the patient through a soft rubber catheter, whenever the pain is severe, and the catheter withdrawn, leaving the medicine to be voided in twenty to thirty minutes; or where he is not able to pass anything from the bladder, the catheter is reintroduced and the medicine allowed to escape. My experience with this treatment has been so satisfactory that I cannot refrain from giving it publicity to the profession."—*Ex.*

